# LC-MS analysis of serum lipidomic and metabolomic signatures in pediatric patients with acute lymphoblastic leukemia

**DOI:** 10.1186/s13052-025-01921-z

**Published:** 2025-03-12

**Authors:** Feiyu Yan, Shengnan Wang, Yilin Wang, Yan Sun, Jing Yang, Lirong Sun, Yekaterina Y. Zaytseva, Pan Deng, Lingzhen Wang

**Affiliations:** 1 Department of Pediatrics Hematology and Oncology, The Affiliated Hospital of Qingdao University, Shandong, 266003 Shandong China; 2 Department of Pharmaceutical Analysis, College of Pharmaceutical Sciences, Soochow University, Suzhou, Jiangsu China; 3 Department of Toxicology and Cancer Biology, University of Kentucky, Lexington, KY USA

**Keywords:** Acute lymphoblastic leukemia, Lipidomics, Metabolomics, Sphingolipids

## Abstract

**Background:**

Acute lymphoblastic leukemia (ALL) is a prevalent hematologic malignancy that primarily affects children. The diagnosis and treatment of pediatric ALL remain challenging. This study aimed to identify differential lipids and metabolites that may hold potential for improving ALL treatment.

**Methods:**

In this retrospective case-control study, serum samples obtained from children with ALL and healthy controls were analyzed. Serum lipidome and metabolome alterations of ALL were analyzed by comparing pediatric patients with ALL with healthy controls based on liquid chromatography high-resolution mass spectrometry analysis of serum lipidomic and metabolomic signatures.

**Results:**

We identified 2,298 lipid features in the serum. Among them, 72 (3.13%) differed significantly in pediatric patients with ALL compared to healthy controls. Notably, sphingolipids (ceramide and sphingomyelin) and phospholipids exhibited the most pronounced changes. Targeted analysis of ceramides revealed significantly elevated levels of Cer 18:0 and Cer 20:0 in the serum of pediatric patients with ALL. Additionally, gut microbial-related lipids (such as sulfonolipids and fatty acid esters of hydroxy fatty acids) showed significant alterations. Metabolomic analysis identified 15 differential metabolites, indicating disrupted nucleotide and amino acid metabolism. Furthermore, the dysregulated lipids and metabolites correlated with various blood indicators, with ceramide and nucleosides positively associated with white blood cell count but negatively correlated with hemoglobin and platelet.

**Conclusion:**

These findings shed light on abnormal molecular signatures contributing to pediatric ALL and may serve as potential biomarker panel for therapy of ALL.

## Introduction

Leukemia is the most common cancer in children and adolescents, accounting for almost one-third of cancers. Approximately three-quarters of leukemia are acute lymphocytic leukemia (ALL), which is characterized by uncontrolled proliferation of abnormal and immature lymphocytes [1]. ALL remains a challenging malignancy despite advances in treatment. Identifying novel drug targets is critical for improving therapeutic outcomes and overcoming the limitations of current treatment options. Metabolic reprogramming has been recognized as an important cancer hallmark, and it is characterized as the ability of cancer cells to alter their metabolism to support tumor growth and angiogenesis, which may be linked to identification of potential drug targets [2].

Metabolomics and lipidomics involve the global analysis of biochemicals, providing crucial insight into metabolic alterations under cancerous conditions. These techniques also hold promise for rationalizing the selection of targeted therapies tailored to the metabolic characteristics of cancer patients [3, 4]. In the context of ALL, metabolomics has been employed to screen potential diagnostic biomarkers during initial treatment. Schraw et al. used liquid chromatography-mass spectrometry (LC-MS) to profile end-induction plasma, bone marrow, and cerebrospinal fluid from children with B-cell acute lymphoblastic leukemia (B-ALL). Notably, there was considerable overlap in metabolomes across these samples, leading to the conclusion that plasma could serve as a suitable matrix for B-ALL biomarkers [5]. Saito et al. conducted plasma metabolomic profiling at initial- and post-induction therapy time points in patients with ALL, detecting 1305 metabolites and lipids. Over 20% of these biochemicals exhibited significant alterations in patients with ALL [6]. Additionally, Bai et al. analyzed serum samples from 15 children with ALL before therapy, along with 30 pediatric patients with ALL in remission and 60 healthy subjects. They identified 30 differentially expressed metabolites between patients with ALL and healthy donors, including 9 lysophosphatidylcholines [7]. While these studies highlight the importance of metabolic disturbances in the systemic circulation (plasma/serum) of patients with ALL, a comprehensive understanding of lipids and metabolites alterations in pediatric patients with ALL, along with their associations with the clinical blood indicators, remains to be fully elucidated.

The aim of this study was to investigate metabolic changes in the serum of pediatric patients with ALL using lipidomic and metabolomic analyses, and explore the associations between the differentially expressed biochemicals and clinical blood test results.

## Materials and methods

### Chemicals and reagents

Ceramide-(d18:1-d7/15:0) and methionine-d3 (≥ 98 atom %D, chemical purity ≥ 99%) were purchased from Sigma-Aldrich (St Louis, MO, USA). Ceramide (Cer) authentic standards (Cer 16:0, 18:0, 20:0, 22:0 and 24:1) were obtained from Aladdin (purity > 99%, Shanghai, China). Bovine serum albumine (BSA, > 95%) was purchased from Sinopharm Chemical Reagent Co., Ltd (Shanghai, China). Cer-(d18:1-d7/15:0) and methionine-d3 were dissolved in methanol and mixed to prepare the internal standard working solution at the final concentration of 10 μg/mL for both compounds. Cer stock solutions (1 mg/mL) were prepared by dissolving an accurately weighed amount of each Cer standard in methanol. HPLC-grade methanol, isopropanol, acetonitrile, and formic acid were used for LC-MS analysis. Other chemicals and solvents were of reagent grade.

### Study population and sample collection

Ten children diagnosed with ALL during 2017–2018 at the Affiliated Hospital of Qingdao University participated in the study. Additionally, ten healthy children without hematological and oncology diseases were included as controls from outpatient clinic. All subjects ranged in age from 0 to 14 years. Samples from patients with ALL were collected prior to treatment. Serum samples were prepared from the collected whole blood and stored at -80^o^C until analysis. Ethical approval was obtained from the Affiliated Hospital of Qingdao University Institutional Review Board (QYFYKYLL 971311920-1), and the study was conducted in accordance with the Declaration of Helsinki. Informed consent was obtained from the parents or guardians of all participating children, and all procedures adhered to relevant guidelines and regulations.

### Clinical blood test

Sysmex hematology analyzer (Sysmex XN9000, Japan) was used to perform blood analysis, including neutrophil count (NEU), white blood cell count (WBC), red blood cell count (RBC), hemoglobin (HGB) and platelet (PLT).

### Untargeted lipidomic and metabolomic analysis

Serum sample preparation was performed following a previously published method [8]. Briefly, a biphasic extraction with water, methanol, and methyl tert-butyl ether (MTBE) was used to separate non-polar and polar metabolites. Ten microliters of internal standard working solution were added to 20 μL of serum sample. After adding 130 μL of methanol, the samples were vortexed for 5 min, and 500 μL of MTBE was added. The samples were shaken for 20 min, and 125 μL of ultrapure water was added to induce phase separation. After centrifugation at 13,000 rpm for 5 min, the upper layer (lipid fraction) was collected, and 400 μL MTBE was added for the secondary extraction. The lipid fraction was pooled and dried under nitrogen flow. The lower layer (aqueous fraction) was collected and lyophilized. The samples were stored at -80^o^C until analysis. Pooled rat plasma was used to prepare quality control (QC) samples, which were extracted as described above.

### Sample preparation for quantitative analysis of ceramides in the serum

Given the endogenous presence of Cer 16:0, 18:0, 20:0, 22:0 and 24:1 in human serum, a surrogate matrix of 5% BSA in water was employed for preparing the calibration standards [9]. Calibration standards of Cer 16:0 (10–3,000 ng/mL), Cer 18:0 (1–3,000 ng/mL), Cer 20:0, 22:0 and 24:1 (3–1,000 ng/mL) were prepared. For calibration standards and QC samples, 20 μL of the standard was added into a 1.5 mL centrifuge tubes, followed by 20 μL of 5% BSA solution and 10 μL of the internal standard solution (Cer-(d18:1-d7/15:0) at 1000 ng/mL). For serum samples, 20 μL of the sample was aliquoted into a 1.5 mL centrifuge tube, and 20 μL of methanol was added, along with 10 μL of the internal standard solution. To each sample, 120 μL of isopropanol was added to precipitate the protein. After vortex mixing and centrifugation at 13,500 rpm for 15 min, the supernatant was analyzed using LC-MS/MS.

### Lipidomic analysis using UHPLC-HRMS

Lipidomic analysis was performed using the SII liquid chromatography system coupled to a Thermo Q-Exactive Focus Orbitrap high resolution mass spectrometer (HRMS) equipped with a heated electrospray ion source (Thermo Scientific, CA, USA). Ultra-high performance liquid chromatography (UHPLC) was performed on a Waters ACQUITY UPLC®BEH-C8 column (2.1 × 50 mm, 1.7 μm). The mobile phase A consisted of 60/40 water/acetonitrile (containing 10 mM ammonium formate and 0.1% formic acid), while mobile phase B consisted of 90/10 isopropanol/acetonitrile (containing 10 mM ammonium formate and 0.1% formic acid). The flow rate was 250 μL/min, and the column temperature was maintained at 40^o^C. The mobile phase gradient was from 32% B to 97% B over 25 min, maintained at 97% B for 4 min, and re-equilibrated with 32% B for 6 min. Lipid fraction samples were dissolved in 100 μL of the initial mobile phase, and 10 μL was injected into UHPLC-HRMS. The MS analysis was performed in both positive and negative ionization modes. The ion source parameters were set as follows: the sheath gas and auxiliary gas were nitrogen, and the flow rate was 35 arb and 12 arb, respectively. The capillary temperature was 330^o^C, and the spray voltage was 4000 V and − 3800 V for positive and negative ionization modes, respectively. The temperature of the probe heater was 325^o^C. The S-Lens RF level was 45. The serum samples were injected following random orders during the analysis. During sample analysis, serum samples were injected in random order, generated using an online random number generator (https://www.calculator.net/calorie-calculator.html). The QC sample was injected after every six serum samples. Method reproducibility was assessed by calculating the median relative standard deviation (RSD) of UHPLC-HRMS peak areas for metabolites across all technical replicates of QC samples.

### Metabolomic analysis using UHPLC-HRMS

Freeze-dried aqueous fraction samples were reconstituted in 100 μL of methanol/water (8: 2, v/v), and 10 μL was injected into UHPLC-HRMS. The metabolomic analysis was performed by using a previously reported method [10]. Briefly, Chromatographic separation was performed on the SeQuant®ZIC®-pHILIC column (2.1 × 150 mm, 5 μm). The mobile phase consisted of (A) 20 mM (NH_4_)_2_CO_3_ aqueous solution containing 0.1% NH_4_OH and (B) acetonitrile. The flow rate was 150 μL/min, the column temperature was maintained at 40^o^C. Chromatographic separation was performed using a linear gradient from 80 to 20% B over 20 min, maintained at 20% B for 1 min, and re-equilibrated at 80% B for 5 min. The MS was performed in both positive and negative ion modes. The ion source parameters were set as follows: the sheath gas and auxiliary gas were nitrogen, and the flow rate was 40 arb and 15 arb, respectively. The capillary temperature was 275^o^C, and the spray voltage was 3000 V and − 3000 V for positive and negative ionization modes, respectively. The temperature of the probe heater was 325^o^C. The S-Lens RF level was 45. Sample injection followed the same sequence as the lipidomic analysis.

### Targeted analysis of ceramides using LC-MS/MS

Quantitative analysis of serum ceramides was performed using a high performance liquid chromatography system (Shimadzu, Japan) coupled to an API4000 Qtrap MS (Sciex, USA). Chromatographic separation was performed on a Venusil® XBP C18 (2.1 × 50 mm, 5 μm) column. Mobile phase A consisted of 60/40 water/acetonitrile (containing 10 mM ammonium formate and 0.1% formic acid) and B consisted of 90/10 isopropanol/acetonitrile (containing 10 mM ammonium formate and 0.1% formic acid). The flow rate was 300 μL/min, and the column temperature was 40^o^C. The mobile phase gradient was 80% B to 99% B over 1.5 min, maintained at 99% B for 2.5 min, and re-equilibrated with 80% B for 3 min. The parameters of mass spectrometry detection were set as follows: collision gas (CAD) was 8 psi; curtain gas (CUR) was 10 psi; heating gas (GS1) was 40 psi; nebulizer gas (GS2) was 40 psi. The ion spray voltage (IS) was 4500 V. The ion source temperature was 450 °C. The data acquisition and analysis were performed using Analyst 1.6.3 (AB Sciex, United States). Calibration curves were established using a 1/x^2^ weighted linear regression. The t-test was used to compare the difference of ceramide levels between pediatric patients with ALL and healthy controls, with significance accepted at *p* < 0.05.

### Lipidomic and metabolomic data processing

The raw LC-MS data (*.RAW) were converted to the *.abf format using abfConverter (Reifycs Inc). These transformed data were then analyzed with MS-DIAL software (v.4.90) for LC-MS peak alignment, identification, and integration. A blank comparison was performed by retaining LC-MS features where the maximum intensity from a serum sample was at least ten times higher than the average of the solvent blanks. For metabolomic data analysis, the MSP libraries of MSMS-Public-Pos-VS16 and MSMS-Public-Neg-VS16 were used, which contain the spectral information of metabolite standards under positive and negative modes, respectively. In lipidomic data analysis, [M + H]^+^, [M + NH_4_]^+^ and [M + H−H_2_O]^+^ were selected in the adducted ion settings for the positive ionization mode data, while [M−H]^−^ and [M + HCOO]^−^ were used for negative ionization mode. The lipid class nomenclature is based on commonly accepted terms and builds upon the LIPID MAPS terminology [11, 12]. Both lipidomic and metabolomic data have been deposited in the EMBL-EBI MetaboLights database with identifiers of MTBLS8814 and MTBLS8817, respectively.

### Statistical analysis

The data normalized with internal standards were imported into MetaboAnalyst (versions 5.0, https://genap.metaboanalyst.ca/) for univariate and chemometrics statistical analysis, including unsupervised principal component analysis (PCA), partial least squares discriminant analysis (PLS-DA) and volcano plot analysis. Differential lipids were selected based on false discovery rate (FDR) adjusted *p*-value < 0.05 and|log2(fold change)| > 1 in the volcano plot. Statistical significance of differential metabolites between ALL and control groups was determined using Student’s t-test in GraphPad Prism 8.0.1 (Boston, MA, USA).

Metabolomic networks were constructed using the MetaMapp approach (web-based portal, version 2020), which calculated biological pathways relevance (KEGG reactant pairs) and chemical structural similarity (Tanimoto coefficient > 0.7). The resultant data were downloaded from the job page and further visualized in CytoScape 3.8.0. Spearman’s correlation analysis was performed on the Tutools platform (http://www.cloudtutu.com/) to explore the relationship between differential lipids/metabolites and clinical blood indicators. The correlation was evaluated using the correlation coefficient (r) value and its 95% confidence interval (CI), expressed as r (lower CI, upper CI), with *p* < 0.05 indicating statistical significance. Heatmaps for the correlation analysis were generated using GraphPad Prism 8.0.1 (Boston, MA, USA).

## Results

### Demographic and clinical characteristics of subjects

Blood/serum samples from 10 patients with ALL (mean age 6.4 ± 3.6 years, range 0.58-14 years, 4 males and 6 females (4 patients for standard risk, 5 patients for inter-mediate risk and 1 patients for high risk) were investigated (Table 1). Additionally, ten age-matched control subjects (mean age 7.2 ± 2.0 years) were included in the study. All participants were Chinese with normal weight and no cases of overweight or obesity. Blood tests showed that NEU, RBC, HGB, and PLT were significantly lower in ALL patients compared with healthy controls. Although there was a trend of higher WBC in ALL patients compared to control subjects, the difference was not statistically significant (Table 1).

**Table 1:** Demographic and clinical characteristics

Demographic characteristics	ALL (*n* = 10)	Con (*n* = 10)	*p*
Age, years	6.4 ± 3.6	7.2 ± 2.0	0.5509
Sex, N (%)			
Male	4 (40)	8 (80)	0.0679
Female	6 (60)	2 (20)	
BMI, N (%)			
< 18.5 kg/m^2^	9 (64.3)	6 (60)	0.2895
18.5–23.9 kg/m^2^	5 (35.7)	4 (60)	
WBC	80.1 ± 140.0	7.1 ± 1 0.8	0.1356
NEU	1.5 ± 1.9	3.4 ± 1.3	0.0229
RBC	3.2 ± 0.8	4.8 ± 0.3	< 0.0001
HGB	89.1 ± 20.4	133.8 ± 6.6	< 0.0001
PLT	78.9 ± 45.5	265.4 ± 36.7	< 0.0001
ALB	39.3 ± 5.3	43.1 ± 2.3	0.0623

Mean ± SD was shown for the variable. WBC, white blood cell count; NEU, neutrophil count; RBC, red blood cell count; HGB, hemoglobin; PLT, platelet; ALB, albumin

### Multivariate analysis of lipidome and metabolome

**Fig. 1:**
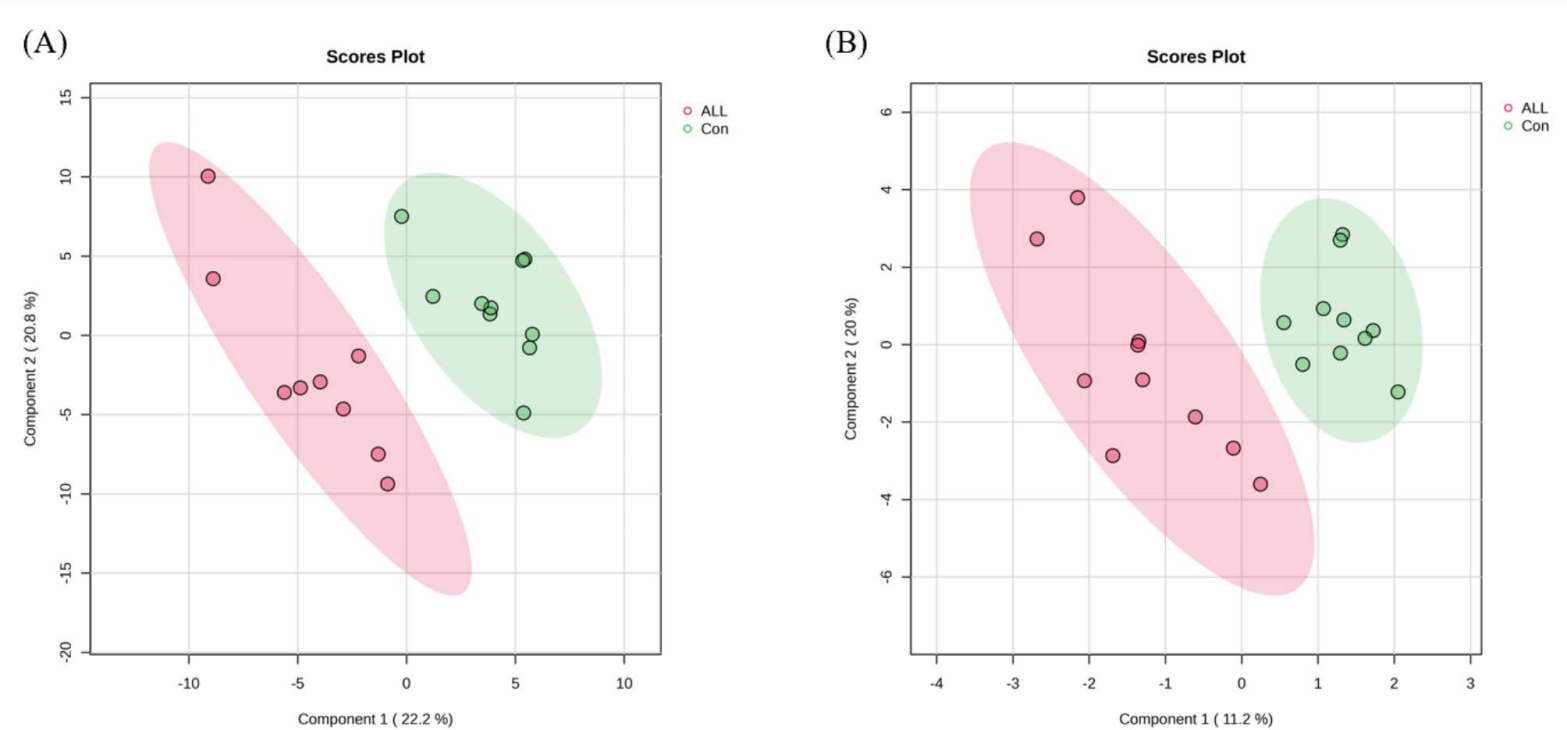
Partial least squares discriminant analysis of lipidome (**A**) and metabolome (**B**) in serum samples from pediatric ALL patients and control subjects. Red circle: ALL sample; green circle: control sample

The median RSD values for lipids and metabolites detected in QC samples were less than 15%, indicating that the analyses were reproducible. Multivariate data analysis, including PCA, PLS-DA, and heatmap visualization, was performed to identify differentially expressed features in pediatric patients with ALL compared to controls. PLS-DA based supervised chemometric algorithm was applied to identify the differential lipids and metabolites (Fig. 1). The results revealed significant differences between patients with ALL and controls, indicating considerable variation in serum lipid and metabolite compositions under the ALL conditions. The total cumulative variance of the first two principal components was 43% (component 1: 22.2%, component 2: 20.8%) for lipidomics (Fig. 1A), and 31.2% (component 1: 11.2%, component 2: 20%) for metabolomics (Fig. 1B), indicating that the model effectively distinguished between the two groups based on biochemical characteristics.

### Lipidomics

**Fig. 2:**
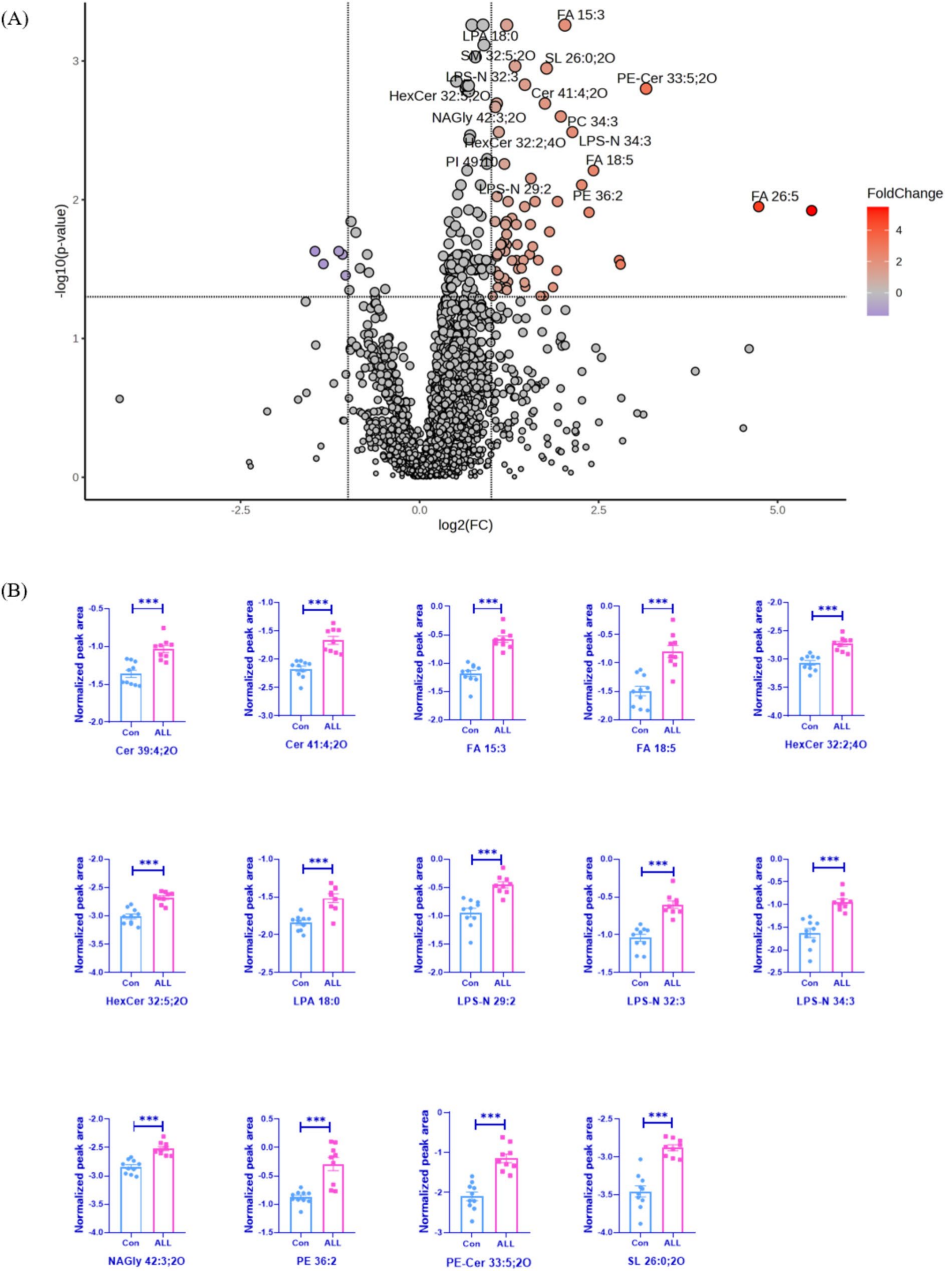
Differentially expressed lipids in serum samples from pediatric ALL patients compared to control subjects (*n* = 10). (**A**) Volcano plot illustrating serum lipid differences between pediatric patients with ALL and controls. Red circle: FDR adjusted *p* < 0.05 and log2(FC) > 1; blue circle: FDR adjusted *p* < 0.05 and log2(FC) < -1. (**B**) Histogram of the 14 top significantly changed lipids (-Log10(p) > 3, Log2(FC) > 1). *** *p* < 0.001; **** *p* < 0.0001

After MS-DIAL data processing, 952 and 1,346 lipids were detected in positive and negative ionization mode, respectively. Changes in serum lipid profiles in patients with ALL are illustrated in the volcano plots (Fig. 2A). A total number of 72 differentially expressed lipids (Log2(FC) > 1 or < 1, FDR adjusted *p* < 0.05) were detected, with 67 lipids increased and 5 decreased in the pediatric ALL group compared to controls. Fourteen lipids from nine lipid classes, including Cer, hexosylceramide (HexCer), fatty acid (FA), lysophosphatidic acid (LPA), N-acyl-lysophosphatidylserine (LNAPS), N-arachidonoyl glycine (NAGly), phosphatidylethanolamine (PE), ceramide phosphoethanolamine (PE-Cer) and sulfonolipid (SL), were identified as the most significantly changed lipid species with log10(p) values > 3. Histograms of these lipids were shown in Fig. 2B.

**Fig. 3:**
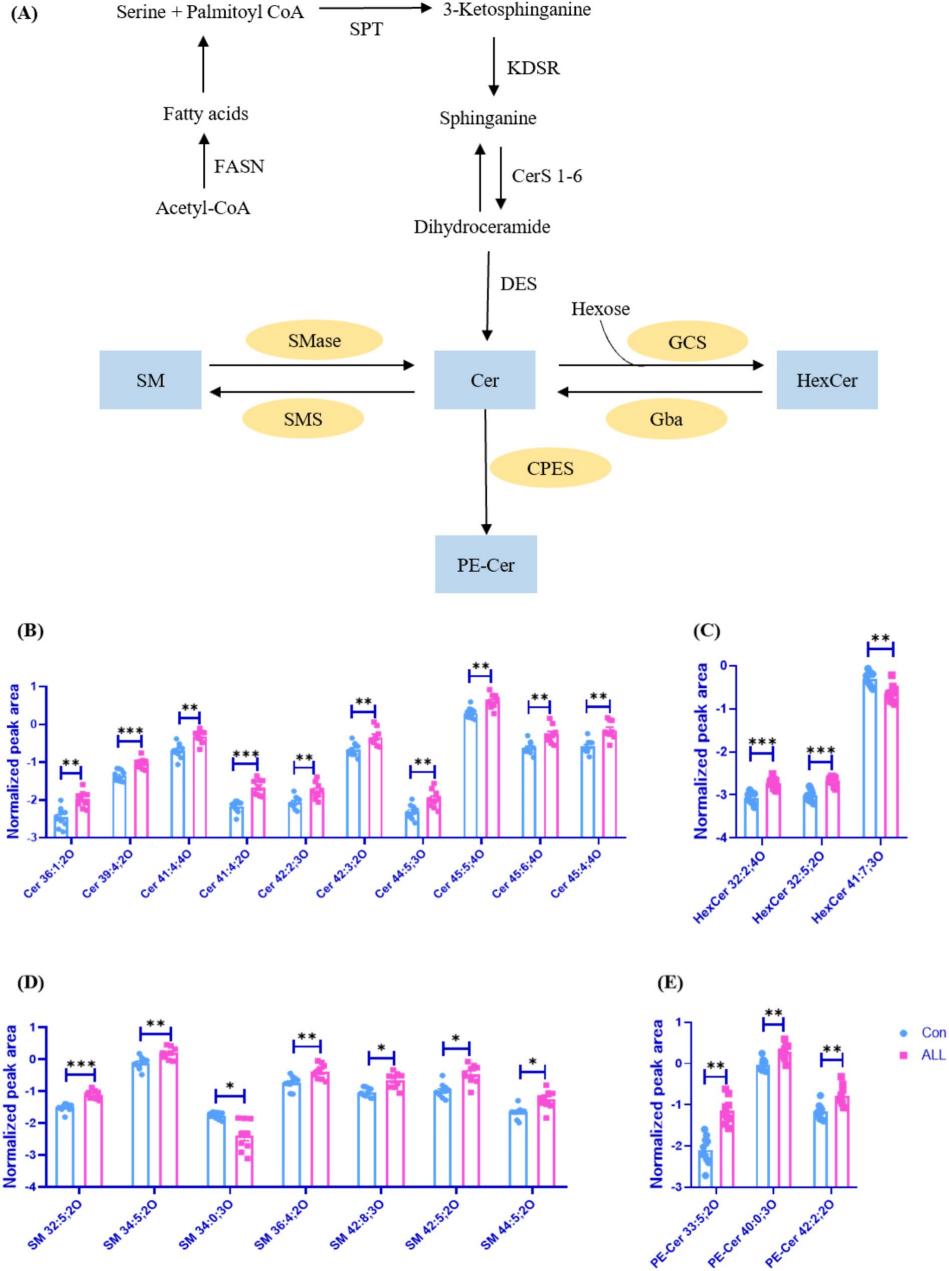
Sphingolipid metabolism disruption in the serum of pediatric ALL patients. (**A**) Metabolic pathways of sphingolipids, including key enzymes FASN (fatty acid synthase), SPT (serine palmitoyltransferase), KDSR (3-keto-dihydrosphingosine reductase), CerS (ceramide synthase), DES (dihydroceramide desaturase), SMase (sphingomyelinase), SMS (sphingomyelin synthase), CPES (ceramide phosphoethanolamine synthase), Gba, (glucocerebrosidase), and GCS (glucosylceramide synthase). The blue box highlighted differential sphingolipids detected in this study, while the orange oval represented the enzymes involved in the metabolism of sphingolipids; Histograms showing differentially expressed (**B**) ceramides, (**C**) sphingomyelin, (**D**) hexosylceramide, and (**E**) ceramide phosphoethanolamine between ALL patients and control subjects. The relative abundance of each lipid was demonstrated by the Log10. * *p* < 0.05; ** *p* < 0.01; *** *p* < 0.001; **** *p* < 0.0001

The 72 differential expressed lipids were primarily clustered in the lipid class (containing more than 5 differentially expressed lipid species) of lysophosphatidylserine (LNAPS), Cer, sphingomyelin (SM), phosphatidylcholine (PC). Sphingolipid was identified as one of the major affected lipid classes in patients with ALL. Among the 72 differential lipids, 26 belonged to the sphingolipid class (36.1%), including Cer, SM, HexCer, PE-Cer, dihexosylceramide (Hex2Cer) and sulfatide (SHexCer). Among these sphingolipids, 88.5% were elevated in the serum of pediatric patients with ALL. The metabolic pathway of sphingolipids was shown in Fig. 3A. Histogram of Cer, HexCer, SM, and PE-Cer differential lipids were shown in Fig. 3B-E.

### Metabolomics

**Fig. 4:**
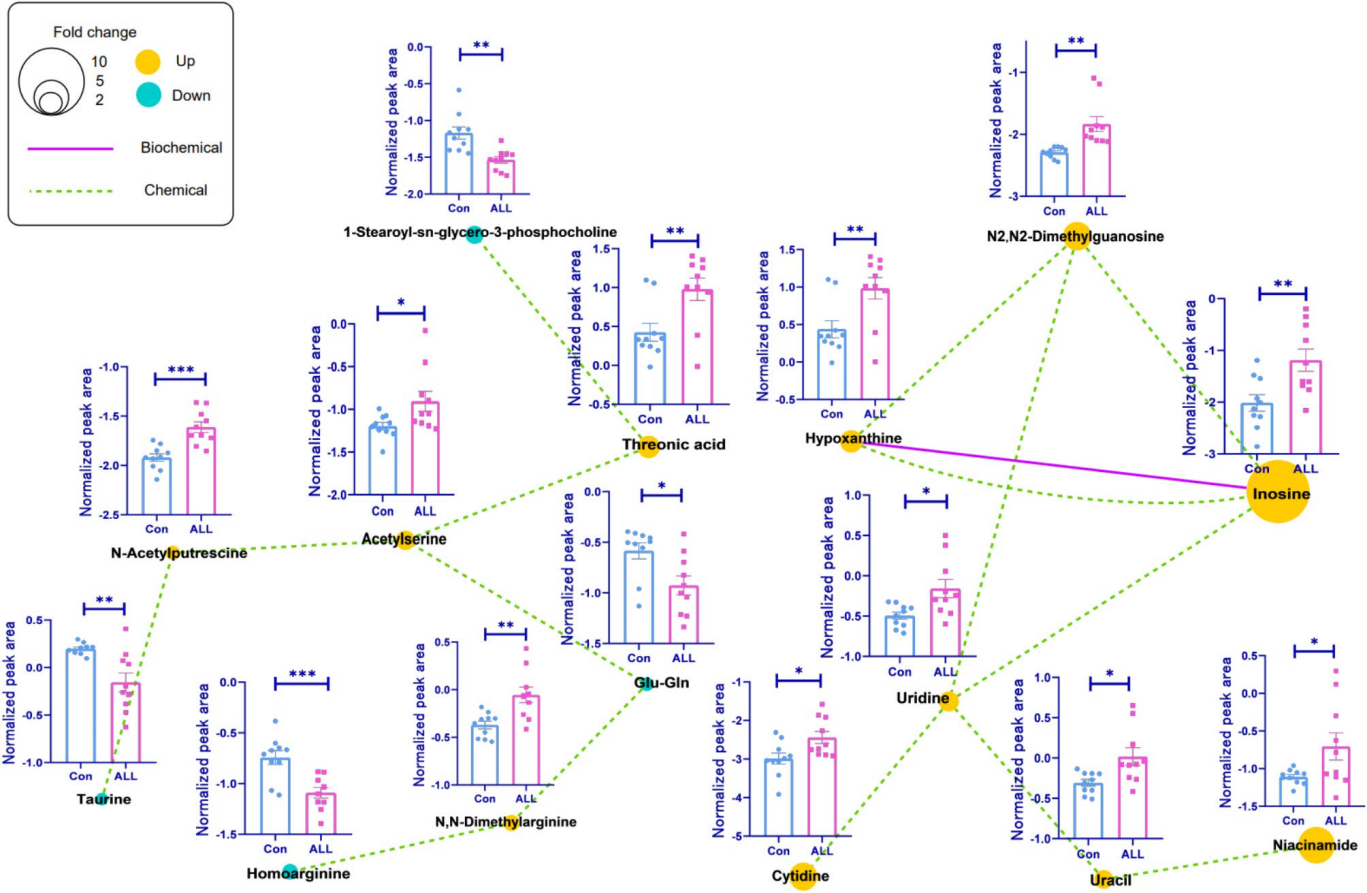
MetaMapp metabolomic networks illustrating differentially expressed metabolites in serum samples from pediatric ALL patients. Nodes represent individual metabolites, while edges denote biochemical (KEGG reactant pairs) and chemical (Tanimoto coefficient > 0.7) relationships. The orange circle indicates a significant increase in pediatric patients with ALL compared to controls, whereas the blue circle represents a significant decrease. The relative abundance of each metabolite is demonstrated by the Log10 (normalized peak area). * *p* < 0.05; ** *p* < 0.01; *** *p* < 0.001

After MS-DIAL processing of metabolomic data, 152 and 137 metabolites were assigned under positive and negative ion modes, respectively. Volcano plot and variable importance in projection (VIP) scores were used to identify differentially expressed metabolites in the serum of patients with ALL. Fifteen differential metabolites were identified with *p* < 0.05 (Table 2). Among these, 11 metabolites were increased and 4 metabolites were decreased in the ALL group compared to the control group. To further delineate the metabolic changes in patients with ALL, a network analysis was performed, resulting two compound clusters covering nucleotides and amino acids (Fig. 4). All metabolites in the nucleotide cluster were elevated in patients with ALL compared with controls. Conversely, decreases in homoarginine, 1-stearoyl-sn-glycero-3-phosphocholine, glu-gln, and taurine were observed in the ALL group compared to control subjects (Fig. 4).

**Table 2:** Differential expressed metabolites for metabolomics analysis

Metabolites	Formula	m/z	RT (min)	*P* value	Log2(FC) (AL/Con)	Adduct
Uracil	C_4_H_4_N_2_O_2_	111.0185	4.93	0.0128	1.4925	[M-H]^−^
Niacinamide	C_6_H_6_N_2_O	123.0555	4.04	0.0410	2.5204	[M + H]^+^
Taurine	C_2_H_7_NO_3_S	126.0219	8.83	0.0026	-0.85337	[M + H]^+^
N-Acetylputrescine	C_6_H_14_N_2_O	131.118	12.73	0.0002	1.0755	[M + H]^+^
Threonic acid	C_4_H_8_O_5_	135.03	5.25	0.0077	1.7548	[M-H]^−^
Hypoxanthine	C_5_H_4_N_4_O	137.0457	5.14	0.0077	1.7431	[M + H]^+^
Acetylserine	C_5_H_9_NO_4_	146.0445	2.38	0.0333	1.5244	[M-H]^−^
Homoarginine	C_7_H_16_N_4_O_2_	189.1347	16.78	0.0007	-1.2178	[M + H]^+^
N, N-Dimethylarginine	C_8_H_18_N_4_O_2_	203.1502	13.38	0.0028	1.2431	[M + H]^+^
Uridine	C_9_H_12_N_2_O_6_	243.0615	4.94	0.0131	1.5601	[M-H]^−^
Cytidine	C_9_H_13_N_3_O_5_	244.0926	6.52	0.0198	2.1003	[M + H]^+^
Inosine	C_10_H_12_N_4_O_5_	267.074	5.77	0.0063	3.3248	[M-H]^−^
Glu-Gln	C_10_H_17_N_3_O_6_	274.1039	10.07	0.0123	-0.98898	[M-H]^−^
N2,N2-Dimethylguanosine	C_12_H_17_N_5_O_5_	312.1301	4.15	0.0015	2.1282	[M + H]^+^
1-Stearoyl-sn-glycero-3-phosphocholine	C_26_H_54_NO_7_P	524.3721	4.05	0.0012	-1.4239	[M + H]^+^

### Targeted analysis of ceramides in the serum

Cer is one of the major types of differential lipids identified in the untargeted lipidomic analysis. Therefore, a targeted analysis of Cer in serum was performed to confirm the lipidomic results. Table 3 showed the serum concentrations of Cer 16:0, 18:0, 20:0, 22:0 and 24:1 in pediatric patients with ALL and healthy children. The serum level of Cer 18:0 was significantly higher in pediatric patients with ALL compared to healthy controls, consistent with the lipidomic result. The UHPLC-Orbitrap MS response of Cer 20:0 was below the intensity limit setting in the MS-DIAL and was therefore filtered out in lipidomic data analysis. However, targeted analysis with improved detection sensitivity revealed that the serum level of Cer 20:0 was significantly higher in the pediatric patients with ALL compared to the controls.

**Table 3:** Serum ceramide levels in ALL pediatric patients and healthy control subjects

Ceramide	ALL (ng/mL)	Con (ng/mL)	*p* value
C16	543.3 ± 483.9	355.2 ± 105.6	NS
C18	158.9 ± 98.5	72.7 ± 35.6	0.0238
C20	525.8 ± 192.4	323.1 ± 111.6	0.0136
C22	1676.0 ± 428.8	1624.3 ± 466.1	NS
C24:1	2080.4 ± 765.2	1517.9 ± 519.9	NS

Data are presented as means ± SD. NS: not significant

### Correlation of differentially expressed serum biochemicals with the clinical blood indicators

**Fig. 5:**
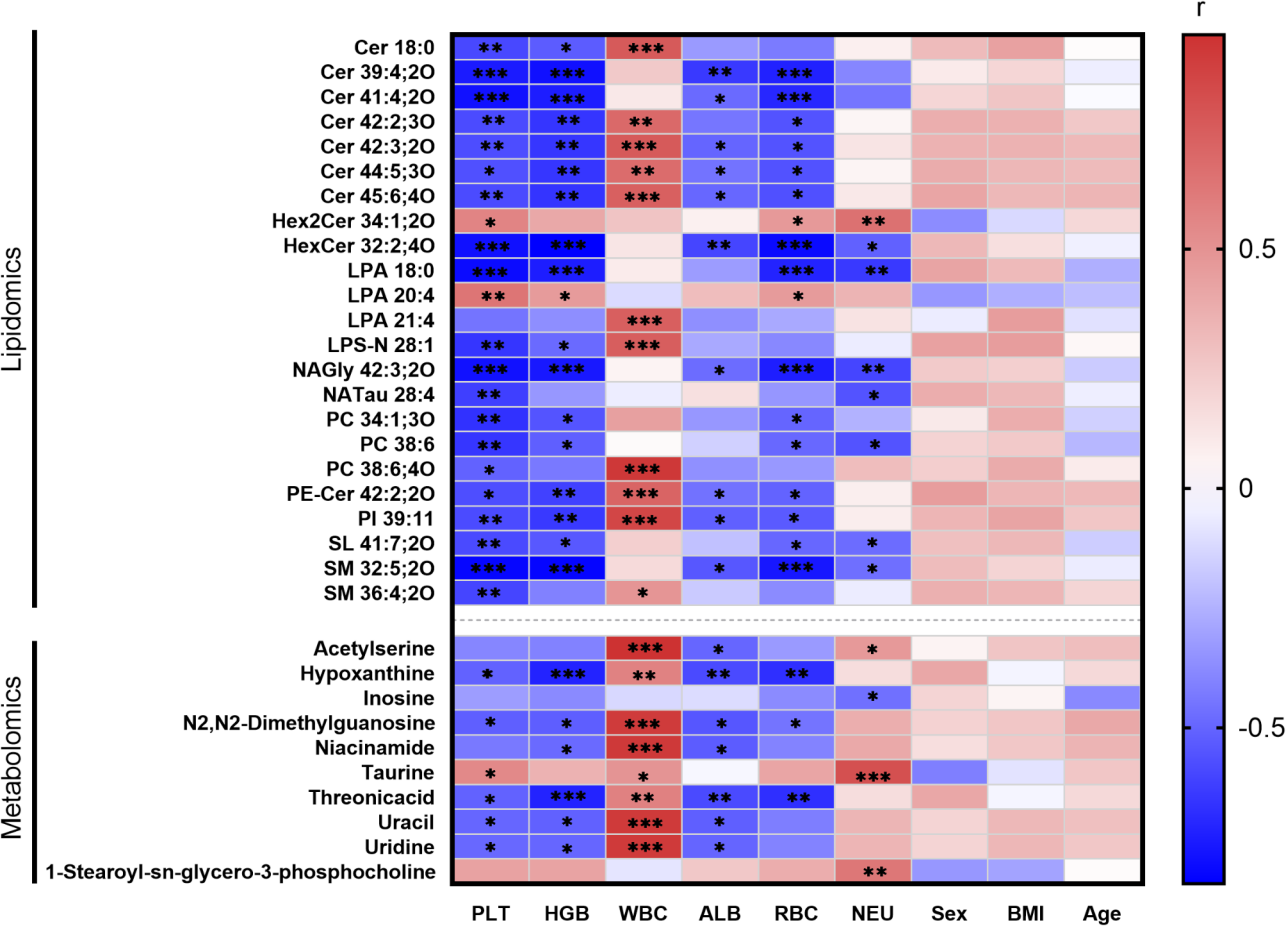
Correlation heat map of differentially expressed lipids and metabolites with clinical blood indicators. The correlation coefficient r represented the correlation between two variables. * *p* < 0.05; ** *p* < 0.01, *** *p* < 0.001

The clinical indicators of RBC, HGB, and PLT were significantly decreased in the blood of patients with ALL (Table 1). A correlation analysis of differentially expressed lipids and metabolites with laboratory clinical indicators was conducted using Spearman’s correlation analysis. The results indicated significant associations between the differential lipids/metabolites and clinical blood indicators (Fig. 5). In addition, these differential lipids/metabolites were not significantly correlated with age, BMI, or sex, while 23 lipids were significantly correlated with blood indicators, including 12 sphingolipids (7 ceramides, 2 hexosylceramides, 2 sphingomyelins, and 1 ceramide phosphoethanolamine) and 8 phospholipids. Eleven lipids and 8 metabolites were positively correlated (*p* < 0.05) with WBC, which showed an increasing trend in the patients with ALL, though not statistically significant (Table 1). The levels of Cer 18:0 was increased in the serum of patients with ALL, and its correlation coefficient (r) with WBC was 0.76 (CI: 0.47, 0.90, *p* = 0.0002). Conversely, the correlation coefficient (r) of Cer 18:0 with HGB and PLT was − 0.53 (CI: -0.79, -0.10, *p* = 0.0199) and − 0.59 (CI: -0.83, -0.19, *p* = 0.0073), respectively. Similarly, nucleosides such as uracil and hypoxanthine were the major metabolites positively associated with WBC but negatively correlated with PLT and HGB.

## Discussion

In this study, the serum samples from pediatric patients with ALL were investigated using lipidomic and metabolomic analyses, and the correlations of differentially expressed biochemicals with clinical blood indicators were identified. Sphingolipid was the top significantly changed lipid class in pediatric patients with ALL, which accounted for 34.0% of the detected differential lipids, and serum sphingolipid levels were significantly higher in the pediatric patients with ALL compared to healthy controls. Targeted Cer analysis showed that Cer 18:0 and Cer 20:0 were upregulated in the serum of pediatric patients with ALL. Additionally, Cer 18:0 was closely correlated with the blood indicators of WBC, HGB and PLT.

Ceramides can be generated by *de novo* synthesis from palmitoyl CoA catalyzed by fatty acid synthase (FASN) [13]. It was reported that FASN was overexpressed in a variety of cancers including leukemia [14, 15]. Additionally, FASN overexpression led to the higher basal ceramide levels in MCF7 cells [16]. Intriguingly, FASN was a poor prognostic factor for pediatric ALL and its upregulation contributed to poor response to chemotherapy in ALL [17]. These results suggested a potential association between high ceramide levels and drug resistance in ALL. In addition, inhibition of fatty acid synthase activity may be a potential strategy for the treatment of leukemia [14]. CerS1-generated Cer 18:0 influences cancer cell survival, apoptosis, and mitophagy [18]. Moreover, it regulates the resistance to imatinib-induced apoptosis in K562 human chronic myeloid leukemia cells [19]. Dany et al. analyzed CerS1 mRNA and its impact on survival using the AML database in the Cancer Genome Atlas Research Network, and found that 10 out of 166 AML patients exhibited upregulated CerS1 mRNA expression, and showed significantly longer overall and disease-free survival compared with those with basal CerS1 mRNA abundance [20]. Ceramides can be also generated from the hydrolysis of sphingomyelin by sphingomyelinases. Kim et al. identified missense mutations in sphingomyelin phosphodiesterase 3 (SMPD3) in the ALL cell lines of CCRF-CEM and MOLT-4. Further nucleotide sequencing of a panel of 33 ALL cell lines showed that 5 samples had SMPD3 gene mutations, providing genetic evidence on the specific role of SMPD3 in ALL [21]. Taken together, sphingolipids may serve as serum biomarkers in pediatric patients with ALL, and targeting enzymes in the sphingolipid metabolism pathway may open up new avenues for improved combination therapies against leukemia drug resistance. However, sample size in this study is limited and larger populations of childhood patients with ALL in prospective cohort studies may help prove the validity of our results. In future studies, we should increase the number of samples, collect bone marrow and peripheral blood samples when ALL patients are diagnosed, and then isolate mononuclear cells to analyze the expression levels of enzymes in FASN and sphingolipid metabolism pathway (including CerS, SMPD, etc.) [17]. Further analysis of the mechanism of ceramide upregulation in ALL patients will open new avenue for ALL treatment.

Emerging evidence has established a bidirectional relationship between gut microbiota and leukemia. Patients with leukemia exhibit decreased intestinal barrier function and disturbances of the intestinal flora [22, 23]. In addition, the gut microbiome significantly influences the onset, progression, prognosis, and treatment of ALL [23]. Studies have reported a higher relative abundance of Bacteroidetes in patients with ALL compared to controls at the time of diagnosis [24, 25]. Bacteroidetes, one of the most abundant gut microbial phyla, can produce sphingolipids [26]. Specifically, biosynthesis of Cer beta-hydroxy fatty acid-sphinganine and PE-Cer have been observed in *Bacteroides fragilis* and *Bacteroides thetaiotaomicron* [27, 28]. Furthermore, recent research has demonstrated that sphingolipids derived from Bacteroidetes could modulate the host ceramide levels [29]. SL, an unusual class of sphingolipids with a sulfonic acid group in the sphingoid base, is structurally related to ceramides and is a lipid class found in the outer membranes of Gram-negative bacteria in the Bacteroidetes phylum. Therefore, gut microbial dysbiosis could partially explain the elevated serum sphingolipids and SL levels in patients with ALL observed in the current study. Taken together, the gut microbiome plays a multifaceted role in blood cancers, and our results suggest a potential role for gut microbial-related lipids in ALL. In follow-up studies, stool samples should be collected at the same time as serum. The addition of disease microbiome analysis, combined with multi-omics analysis, will provides more evidence for the link between microbiota and lipid profiles.

Cancer cells are known to reprogram nucleoside and amino acid metabolism to sustain tumor progression, support relapse, and confer the resistance to anticancer drug [30]. The current metabolomic study showed that nucleoside and amino acid metabolism were disturbed in patients with ALL, with higher levels of uracil, hypoxanthine, uridine, cytidine, inosine, threonic acid and homoarginine observed in children with ALL compared to controls. Consistent with our findings, Hashimoto et al. reported that plasma hypoxanthine levels in patients with ALL (25.5 ± 17.5 μM) were significantly higher than those in healthy adult controls (4.0 ± 1.4 μM) [31]. Nucleotide biosynthesis is a fundamental metabolic process crucial for cell proliferation and survival, as it ensures the availability of raw material for nucleic acid synthesis. Rapid proliferating cancer cells must meet the high nucleotide demand associated with their growth [32]. While the abnormal metabolism of tumors promotes cell proliferation, it also introduces metabolic vulnerabilities that can be therapeutically targeted. Nucleotide synthesis pathways have been effectively targeted in leukemia [33–36]. For instance, reducing the production of deoxycytidine triphosphate by inhibiting both *de novo* and nucleotide rescue pathway has shown efficacy in mouse models of ALL [36]. Furthermore, dysregulated nucleotide metabolism can interact with the host immune system and promote tumor growth. Increasing evidence suggests that targeting nucleotide metabolism can increase the antitumor immune response [37]. Current pediatric ALL treatment strategies have improved 5-year overall survival rates to exceeding 90% [38]. However, a considerable percentage of patients suffer from relapse, and the survival rates drop to about 50% in relapsed cases [39]. Since relapse is mainly due to chemotherapy resistance, therapies that may synergize with or re-sensitize cells to chemotherapy are of urgent need. Combining nucleotide-targeted therapies with existing chemotherapy regimens may enhance treatment efficacy, and investigating these synergistic effects could lead to more effective therapeutic strategies.

The current study has limitations: (1) The sample size is small which limits the generalizability of our findings; (2) The potential mechanisms underlying the observed upregulation of serum ceramide levels in ALL patients are not investigated; (3) The diversity and composition of the microbiota at the time of ALL diagnosis were not analyzed, and the potential connection between the microbiota and lipid profiles was not established. These limitations underscore the need for further investigation. To address these gaps, we propose future studies with larger cohorts to improve the robustness of our findings. We also plan to collect bone marrow and peripheral blood samples at the time of ALL diagnosis for isolating monocytes and extracting total RNA. This will enable detailed analysis of the expression levels of key enzymes involved in the FASN and sphingolipid metabolism pathways (e.g., CerS, SMPD, etc.), providing insights into the mechanisms of ceramide upregulation in ALL patients. Moreover, stool samples will be collected concurrently with serum samples in future experiments. By incorporating microbiota diversity analysis and leveraging multi-omics approaches, we aim to uncover potential links between microbiota composition and lipid profiles.

## Conclusions

In conclusion, comprehensive lipidomic and metabolomic analyses were used to identify biochemical signatures in the serum of pediatric patients with ALL. Sphingolipid was identified as a major lipid group significantly upregulated in these patients, showing a positive association with WBC. Additionally, lipids potentially related to a disrupted gut microbiome were identified in ALL subjects. Metabolomic analysis also revealed the alterations in nucleoside and amino acids metabolism in children with ALL. Further large-scale clinical studies are necessary to confirm these biochemical changes and their association with gut microbiome functions in pediatric ALL patients.

## Data Availability

Lipidomic and metabolomic data have been deposited to the EMBL-EBI MetaboLights database with the identifiers of MTBLS8814 and MTBLS8817, respectively.
